# Local Structure and Dynamics in Solvent‐Free Molten Salt ${\mathrm{Ca}}^{2+}$‐Electrolytes

**DOI:** 10.1002/cphc.202500090

**Published:** 2025-06-24

**Authors:** Carolina Cruz, Patrik Johansson

**Affiliations:** ^1^ Department of Physics Chalmers University of Technology 41296 Gothenburg Sweden; ^2^ Alistore‐ERI CNRS FR 3104 15 Rue Baudelocque 80039 Amiens France; ^3^ Department of Chemistry– Ångström Uppsala University SE‐751 21 Uppsala Sweden

**Keywords:** cage effect, calcium batteries, dynamic heterogeneity, molecular dynamics simulations, molten salts

## Abstract

Calcium batteries (CaBs) fundamentally offer a promise of sustainable high energy density storage. However, the development of functional CaB electrolytes remains a key challenge. Here, molecular simulations are used to investigate structural and dynamic properties of solvent‐free molten salt electrolytes (MSEs) containing ${\text{Ca}}^{2+}$ and alkali cations (${\text{Li}}^{+}$, ${\text{Na}}^{+}$, $K^{+}$), paired with either FSI or TFSI anions. Two equimolar MSEs, [Li, Na, K, Ca]FSI and [Li, Na, K, Ca] TFSI, are examined across a range of temperatures to better understand cation–anion interactions, coordination and local structure, and ion mobility, in particular with respect to ${\text{Ca}}^{2+}$. The interplay between cation charge density, anion structure, and thermal effects provides valuable insights into the MSEs’ macroscopic behavior. These insights inform the design of advanced electrolytes that enhance ${\text{Ca}}^{2+}$ mobility, supporting the development of next‐generation CaBs.

## Graphical abstract

Molecular dynamics simulations reveal cation caging effects and dynamic heterogeneity in multicomponent molten salt electrolytes. Charge‐to‐radius ratios and anion features influence calcium‐ion mobility, offering pathways to optimize solvent‐free calcium battery performance.

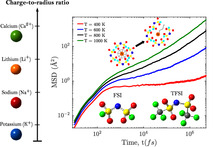

## 1 Introduction

Multivalent metal batteries hold significant potential for energy storage, offering high specific and volumetric capacities and leveraging on the abundance of multivalent metals such as calcium (Ca), magnesium (Mg), and aluminum (Al) in the Earth's crust. These metals are particularly attractive due to their ability to transfer multiple electrons per ion, enabling higher theoretical energy densities compared to monovalent systems. The combination of these properties makes multivalent batteries an appealing choice for sustainable energy storage solutions beyond lithium‐ion batteries (LIBs).^[^ ^1^, ^2^ ^]^

Mg batteries, for instance, benefit from the small ionic radius of ${\text{Mg}}^{2+}$, allowing for high packing densities and energy storage potential.^[^ ^3^, ^4^, ^5^ ^]^ In contrast, calcium's larger ionic radius results in weaker electrostatic interactions and lower coordination barriers, making ${\text{Ca}}^{2+}$ more favorable for fast ion transport.^[^ ^6^, ^7^, ^8^ ^]^ Finally, aluminum offers exceptionally high theoretical capacities due to its trivalent state (${\text{Al}}^{3+}$).

Despite the unique advantages of multivalent metals for battery applications, all three technologies face significant hurdles, particularly in developing functional electrolytes. For Mg batteries, the strong Coulombic interactions of ${\text{Mg}}^{2+}$ reduce ion mobility and limit compatibility with common solvents.^[^ ^4^, ^5^ ^]^ Ca battery (CaB) electrolytes suffer from low ionic conductivity and limited electrochemical stability.^[^ ^1^, ^9^, ^10^, ^11^ ^]^ Al batteries face additional difficulties due to the highly reactive nature of ${\text{Al}}^{3+}$, causing side reactions and inefficient electrodeposition.^[^ ^12^ ^]^

CaBs have gained particular attention due to the promise of high energy density and a standard reduction potential that is close to lithium, making them a strong candidate for medium‐to‐high voltage electrochemical cells.^[^ ^1^, ^2^, ^13^, ^14^, ^15^ ^]^ As compared to ${\text{Mg}}^{2+}$ and ${\text{Al}}^{3+}$ electrolytes, ${\text{Ca}}^{2+}$ also exhibits weaker electrostatic interactions, facilitating ion transport and reducing activation barriers.^[^ ^1^, ^15^ ^]^

Today, CaB liquid electrolytes are made by dissolving calcium salts such as calcium tetrafluoroborate (${\text{Ca}({\text{BF}}_{4})}_{2}$), calcium borohydride (${\text{Ca}({\text{BH}}_{4})}_{2}$), or calcium bis(trifluoromethanesulfonyl)imide (${\text{Ca}}_{{\left(\text{TFSI}\right)}_{2}}$) in organic solvents such as ethylene carbonate, dimethyl carbonate, and glymes.^[^ ^3^, ^16^ ^]^ However, as compared to state‐of‐the‐art LIB electrolytes, these often exhibit lower ionic conductivities and reduced electrochemical stability, leading to worse performance.^[^ ^3^, ^17^ ^]^ Additionally, the flammability of organic solvents in any liquid electrolyte always presents safety risks. Therefore, altogether, there is a growing interest in developing solvent‐free electrolytes that address the performance, stability, and safety concerns.

In this context, molten salt electrolytes (MSEs) are compelling candidates for CaBs due to both offering a quite different concept, as well as their high ionic conductivities and broad electrochemical stability windows.^[^ ^18^, ^19^, ^20^ ^]^ Furthermore, multicomponent MSEs can have low melting points, which prevents the risk of solidification during operation and ensures reliable battery performance.^[^ ^18^, ^21^, ^22^, ^23^ ^]^ Moreover, the absence of solvents offers several advantages, including the elimination of side reactions, gas formation, electrolyte degradation, and electrode damage.^[^ ^18^, ^24^ ^]^ Additionally, direct cation–anion interactions promote unique ion transport mechanisms by bypassing desolvation barriers. However, the molecular‐level understanding of how temperature, ion dynamics, and cation–anion interactions govern ${\text{Ca}}^{2+}$ mobility in multicomponent MSEs remains largely unexplored. MSEs are typically prepared by mixing dry salts in the desired molar ratios, followed by heating the mixture above the eutectic melting point to yield a homogeneous ionic melt. A full description of the experimental preparation protocol, including salt handling and mixing, is available in our previous work.^[^ ^24^ ^]^

Here, MSEs composed of alkali cations and ${\text{Ca}}^{2+}$ with Bis(trifluoromethanesulfonyl)imide (TFSI) or Bis(fluorosulfonyl)imide (FSI) anions are in focus. LiTFSI and LiFSI are salts widely used in lithium‐based battery electrolytes, with LiTFSI being valued for its thermal stability and high‐voltage compatibility, and LiFSI for its high ionic conductivity and lower melting point.^[^ ^25^ ^]^ Similarly, the corresponding sodium (Na) and potassium (K) salts have been studied in various solvent systems. For instance, NaTFSI in alkyl carbonate solvents exhibits a viscosity *≈*0.8 mPa·s lower than the analoguous ${\text{NaPF}}_{6}$ electrolyte 283 K, increasing to 1.2 mPa·s at 298 K.^[^ ^26^ ^]^ On the other hand, KFSI‐based electrolytes in dimethoxyethane (DME) demonstrate higher ionic conductivities, reaching up to 13.6 mS/cm, whereas ${\text{KPF}}_{6}$‐based electrolytes show significantly lower conductivities, primarily due to limited solubility in DME, which hinders ion dissociation.^[^ ^27^ ^]^ While these findings highlight the advantages of FSI‐ and TFSI‐based electrolytes for enhanced ion transport in liquid electrolytes, the behavior of sodium and potassium salts in MSEs remains underexplored. ${\text{Ca(TFSI)}}_{2}$ and ${\text{Ca(FSI)}}_{2}$ are likewise promising candidates for CaBs.

Compared to other common calcium salts such as ${\text{Ca}({\text{BF}}_{4})}_{2}$ ^[^ ^28^, ^29^ ^]^ ${\text{Ca}({\text{BH}}_{4})}_{2}$ ${\text{Ca}({\text{ClO}}_{4})}_{2}$, the bis(sulfonyl)imide‐based salts ${\text{Ca(TFSI)}}_{2}$ and ${\text{Ca(FSI)}}_{2}$ offer improved performance characteristics for battery applications. These include higher electrochemical stability windows,^[^ ^30^ ^]^ better solubility in non‐aqueous solvents,^[^ ^31^ ^]^ and more favorable compatibility with calcium metal anodes.^[^ ^32^ ^]^ Furthermore, both salts are known to form more stable and conductive solid electrolyte interphases (SEIs), which are essential for long‐term battery performance.^[^ ^10^, ^32^ ^]^ This makes ${\text{Ca(TFSI)}}_{2}$ and ${\text{Ca(FSI)}}_{2}$ strong candidates for advanced CaB electrolyte design.^[^ ^30^, ^31^, ^32^ ^]^ Our recent experimental study demonstrated that multicomponent MSEs based on these salts exhibit enhanced ionic conductivity due to increased configurational entropy, which reduces their melting points.^[^ ^24^ ^]^ The study also provided detailed insights into the local coordination environments of ${\text{Ca}}^{2+}$ ions, showing how specific cation‐anion interactions influence the overall physicochemical properties of the electrolytes.

Challenges related to compatibility with calcium metal anodes and achieving stable calcium plating and stripping are critical issues for CaBs. In solvent‐based electrolytes, solvents play a significant role in stabilizing the ${\text{Ca}}^{2+}$ ion at the electrode interface,^[^ ^33^, ^34^ ^]^ but in solvent‐free MSEs, this stabilization must be achieved through direct cation‐anion interactions. Addressing these challenges requires detailed insights into the structural and dynamic properties of MSEs to uncover how multicomponent ion interactions influence transport properties under varying thermal conditions.

Overall, the macroscopic properties of MSEs are governed by their molecular structure and ion dynamics, making a molecular‐level understanding crucial.^[^ ^35^ ^]^ Combining multiple salts leads to more complex and diverse solvation due to a significant variance in the local interactions, stemming from strong Coulombic interactions, which has also been shown to be beneficial for ion transport and cation transfer at the electrolyte/electrode interface.^[^ ^36^, ^37^ ^]^

**Figure 1:**
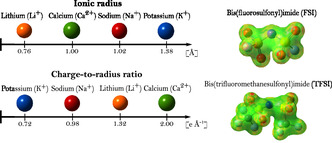
Comparison of ionic radii and charge‐to‐radius ratios for ${\text{Li}}^{+}$, ${\text{Na}}^{+}$, $K^{+}$, and ${\text{Ca}}^{2+}$ cations, alongside the electrostatic potential surfaces of the FSI and TFSI anions, making up the MSEs.

In this work, we investigate two MSEs composed of equimolar compositions of [Li, Na, K, Ca]FSI and [LiNaKCa]TFSI, thus differing only in the choice of the anion (**Figure** **1**). Using classical molecular dynamics (MD) simulations, we elucidate the bulk electrolyte local structure and the cation dynamics, and in particular, the behavior of ${\text{Ca}}^{2+}$. We also examine ion caging effects^[^ ^35^, ^38^ ^]^ and dynamic heterogeneity^[^ ^39^, ^40^ ^]^ at different temperatures. We stress that the applied temperatures are not “real” in the sense of representing intended operating temperatures for CaB, but rather should be viewed as a way to probe the mechanisms at play in a proof‐of‐concept manner. Indeed, in reality, the anions may decompose at these temperatures (but they cannot in the simulations, because the applied method prevents such decomposition). The investigated phenomena are particularly relevant for highly concentrated and solvent‐free electrolytes, as ions in all such electrolytes may become temporarily trapped by surrounding neighbors. Furthermore, any resulting dynamic heterogeneity may create regions of restricted ion movement alongside zones of higher mobility, directly impacting ionic conductivity by reducing the effective number of mobile charge carriers and causing fluctuations in the overall transport properties.^[^ ^41^ ^]^

## 2 Results and Discussion

We start by examining the local structure of the MSEs through analysis of the coordination of each cation using the radial distribution functions (RDFs) and the coordination numbers (CNs) to assess how variations in cation size and charge density influence their interactions with the anions. We also explore temperature‐dependent changes, highlighting the effects on the overall structure and dynamics.

Finally, we study the diffusion and energy barriers for ion motion, and specifically the mechanisms by which cations may escape their cages, including how relaxation processes and barriers evolve with temperature. Altogether, this offers a comprehensive view of cation (local) structure dynamics within the MSEs.

### 2.1 Local Structure

**Figure 2:**
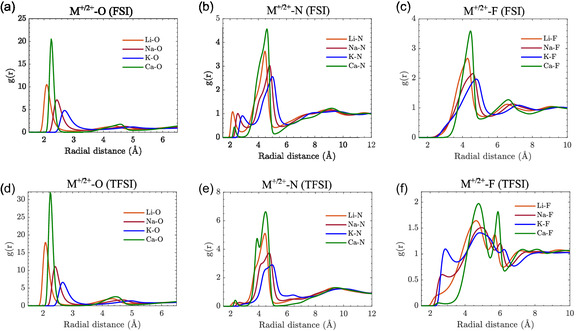
Radial distribution functions, g(r), for $M^{+\text{/2}+}$‐O, $M^{+\text{/2}+}$‐N, and $M^{+\text{/2}+}$‐F interactions in a–c) FSI‐based and TFSI‐based d–f) MSEs.

The local structure analysis of the 400 K simulation RDFs of cation‐oxygen ($M^{+\text{/2}+}$‐O) and cation‐nitrogen ($M^{+\text{/2}+}$‐N) show that, for both FSI and TFSI anions, the former RDFs have a prominent peak 1.8 Å <r< 3.0 Å (**Figure** **2**a,d). The former RDFs converge to unity beyond this range, indicating short‐range order (SRO) and long‐range disorder (LRD) typical of liquids, while for the latter RDFs, a weaker feature is present (Figure 2b and e), which also decays, but at longer distances. The maxima shift to greater radial distances as the ionic radius increases, following the order: ${\text{Li}}^{+}$ < ${\text{Ca}}^{2+}$ < ${\text{Na}}^{+}$ < $K^{+}$.

In the FSI‐based MSE, there are $M^{+\text{/2}+}$‐F features at longer distances, indicating weak interactions and/or simple geometric consequences of the stronger interactions (Figure 2c). In contrast, the TFSI‐based MSEs show additional peaks for ${\text{Na}}^{+}$‐F and $K^{+}$‐F at shorter distances (Figure 2f), likely due to geometrical constraints imposed by the bulkier TFSI and its trifluoromethyl (${\text{CF}}_{3}$) groups. Similar RDF behaviors are observed across all temperatures (SI†).

**Figure 3:**
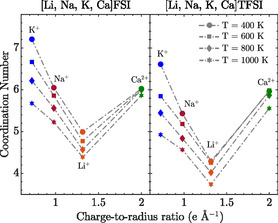
CN as a function of charge‐to‐radius ratio for ${\text{Li}}^{+}$, ${\text{Na}}^{+}$, $K^{+}$, and ${\text{Ca}}^{2+}$ in (left) FSI‐ and (right) TFSI‐based MSEs at 400, 600, 800, and 1000 K.

By integrating up to the first minima in the $M^{+\text{/2}+}$‐O and $M^{+\text{/2}+}$‐N RDFs, we obtain the CNs (**Figure** **3**). In both FSI‐ and TFSI‐based MSEs, the CNs generally increase as the ion size increases, suggesting that smaller cations form stronger interactions with the anions, as expected, leading to tighter packing with fewer anions.

In contrast, larger cations, such as $K^{+}$, with a lower charge‐to‐radius ratio, have higher CNs due to weaker and more diffuse electrostatic interactions, but geometrically, this allows for more anions to fit in the first cation solvation shell. Similar behavior has been reported for KFSI in tetraethylglyoxal (TEG), where at higher concentrations, potassium ions form complexes with CNs reaching up to 7.^[^ ^42^ ^]^ ${\text{Ca}}^{2+}$, despite its relatively small ionic radius, shows high CNs by virtue of its multivalent nature and, hence, strong Coulombic interactions. This is consistent with the findings from Katz et al., who reported that ${\text{Ca}}^{2+}$ ions in crystal structures typically bind to oxygen atoms with preferred CNs ranging from 6 to 8.^[^ ^43^ ^]^ In TFSI‐based electrolytes, the CN of ${\text{Ca}}^{2+}$ is typically reported as 6 in organic solvents, while a combined Density Functional Theory (DFT) and Conductor‐like Screening Model for Realistic Solvation (COSMO‐RS) study identified an energetically preferred CN of 8 in dimethylformamide (DMF)‐based systems.^[^ ^32^ ^]^

While no direct experimental data are currently available for the specific multicomponent MSEs investigated here, our simulation results are consistent with experimental observations in related systems. For instance, Wang et al. used Raman spectroscopy and MD simulations to study ${\text{Ca(TFSI)}}_{2}$ in glyme solvents and reported CNs ranging from 6 to 8, depending on solvent chain length and salt concentration.^[^ ^31^ ^]^ Similarly, Forero–Saboya et al. reported strong ${\text{Ca}}^{2+}$–anion interactions in concentrated TFSI‐based electrolytes using spectroscopic techniques.^[^ ^32^ ^]^ The consistency between our simulated CNs and these experimentally observed values supports the validity of the simulation model and reinforces the structural trends identified in this work.

Across all cations, the TFSI‐based MSE (Figure 3, right) consistently shows lower CNs, i.e., tighter coordination shells, compared to the FSI‐based MSE (Figure 3, left), likely due to steric hindrances. Specifically for the calcium ions, this aligns with Jeong et al., who identified calcium coordinating a single anion as the most stable species in their TFSI‐based systems.^[^ ^44^ ^]^

At higher temperatures (600, 800, and 1000 K), the CNs decrease for all cations in both MSEs, by weakened cation–anion interactions, as expected, and this is more pronounced for the cations with lower charge‐to‐radius ratios, such as $K^{+}$. ${\text{Ca}}^{2+}$, however, maintains relatively stable CNs due to its stronger electrostatic interactions.

In contrast to the CN, the solvation number (SN) is more complicated to determine due to the partial overlap between the first and second solvation shells in the $M^{+\text{/2}+}$‐N RDFs (Figure 2b,e), which may be a result of communal solvation, as reported for highly concentrated electrolytes (HCEs);^[^ ^45^ ^]^ as in HCEs or solvent‐free electrolytes, such as MSEs, the anions are likely to coordinate to more than one cation. This impacts not only the overall solvation but also its interpretation, including the cation–anion interactions, as the concept of solvation shells partly breaks down for MSEs.

**Figure 4:**
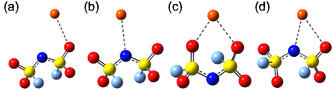
Coordination modes of the FSI anion with a ${\text{Li}}^{+}$ cation: a) monodentate coordination via an oxygen atom (O), b) monodentate coordination via a nitrogen atom (N), c) bidentate coordination via two oxygen atoms (O,O), and d) bidentate mixed coordination via one oxygen and one nitrogen atom (O,N).

Nevertheless, we can hypothesize SNs based on the CNs and by considering the possible coordination modes; both FSI and TFSI can coordinate either monodentately or bidentately, and either by the oxygen atoms or the nitrogen atom, i.e., (O), (N),(O,O), or (O,N)^[^ ^46^, ^47^ ^]^ (**Figure** **4**).

Typically, the SNs are slightly lower than or close to the CNs, and thus monodentate coordination strongly dominates. For somewhat similar electrolytes, Borodin et al. employed MD simulations on ionic liquids doped with LiTFSI and demonstrated that ${\text{Li}}^{+}$ cations are predominantly coordinated by four oxygen atoms, each contributed by distinct TFSI anions, thus monodentate coordination.^[^ ^48^ ^]^ Similarly, Li and coworkers conducted MD simulations on ionic liquids mixed with LiTFSI, revealing a coexistence of monodentate and bidentate coordination modes, where at higher salt concentrations, the prevalence of bidentate coordination diminishes and monodentate coordination increases.^[^ ^49^ ^]^ In contrast, Lassègues et al.^[^ ^50^ ^]^ reported that for LiTFSI‐doped ionic liquids, bidentate coordination dominates at low to moderate salt concentrations. The SI† provides details on all the possible cation coordinations based on the CNs and SNs for each MSE.

**Figure 5:**
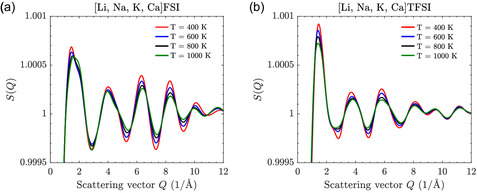
Static structure factor S(Q) as a function of scattering vector *Q* in a) FSI‐ and b) TFSI‐based electrolytes at 400, 600, 800, and 1000 K.

Finally, the static structure factor S(Q) shows stronger cation–anion correlations with sharper, higher peaks for the FSI‐based MSE (**Figure** **5**a), suggesting a more defined SRO as compared to the TFSI‐based MSE (Figure 5b). As the temperature is increased, the peak intensities decrease for both MSEs, indicating reduced SRO. The more pronounced effects for the former MSE suggest its stronger initial correlations to be more sensitive to thermal fluctuations.

Overall, this suggests that the FSI anions might be more effective at “caging” cations, potentially reducing their mobility, which is our next target of investigation.

### 2.2 Dynamics

**Figure 6:**
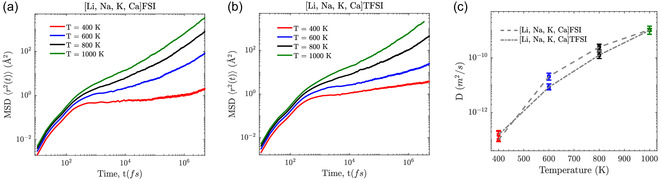
MSDs of a) FSI‐ and b) TFSI‐based MSEs and c) Self‐diffusion coefficients for both MSEs at 400, 600, 800, and 1000 K.

The MSDs illustrate ion mobility trends in the [Li, Na, K, Ca]FSI and [Li, Na, K, Ca]TFSI MSEs across four temperatures: 400, 600, 800, and 1000 K (**Figure** **6**a,b). At 400 K, both MSEs exhibit a plateau, indicating a caging effect that restricts ion mobility, as the limited thermal energy available cannot overcome the strong Coulombic interactions, thus maintaining tight coordination “shells” around the cations. The caging effect is especially pronounced in the FSI‐based MSE, most likely due to less steric hindrance and higher CNs as compared to the TFSI‐based MSE (Figure 3). This behavior aligns with the findings of Biria et al. who demonstrated that mobility decreases with increasing ion pairing and coordination, particularly in ${\text{Ca}}^{2+}$ systems.^[^ ^6^ ^]^ As temperature is increased, the MSDs increase as well, and the plateau diminishes, indicating enhanced ion mobility; ions may escape their cages and diffuse more freely.

The total self‐diffusion provides further insight, and here the FSI‐based MSE outperforms the TFSI‐based MSE, especially for the intermediate temperatures (Figure 6c). At 400 K, much of the ion motion is arrested. At higher temperatures, the difference is less due to the reduced impact of steric hindrances.

**Figure 7:**
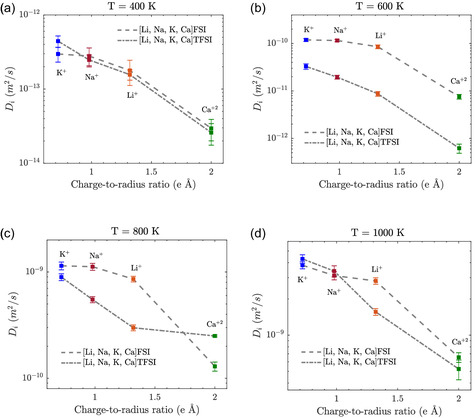
Self‐diffusion coefficients of each cation (${\text{Li}}^{+}$, ${\text{Na}}^{+}$, $K^{+}$, and ${\text{Ca}}^{2+}$) as a function of charge‐to‐radius ratio at a) 400, b) 600, c) 800, and d) 1000 K for both FSI‐ and TFSI‐based MSEs.

Analyzing the individual cation mobilities (**Figure** **7**), the cations with higher charge‐to‐radius ratios, such as ${\text{Ca}}^{2+}$, consistently exhibit slower diffusion across all temperatures and again more pronounced in the FSI‐ based MSE with its tighter coordination “shells”.

From 400 K to 1000 K, all cations exhibit increased self‐diffusion, and the mobility hierarchy remains the same: $K^{+}$ > ${\text{Na}}^{+}$ > ${\text{Li}}^{+}$ > ${\text{Ca}}^{2+}$. At elevated temperatures, the FSI‐based MSE demonstrates notably higher diffusion across all cations. This observation aligns with insights from simulations of MSs, where cations with higher charge‐to‐radius ratios exhibit reduced mobility due to stronger Coulombic interactions and longer ion‐pair lifetimes.^[^ ^51^ ^]^

**Figure 8:**
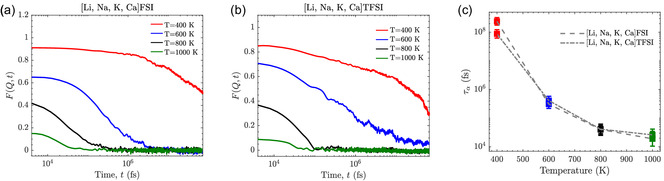
Intermediate scattering structure factor, F(Q,t), for a) [Li, Na, K, Ca] FSI and b) [Li, Na, K, Ca] TFSI MSEs and c) relaxation time extracted from the KWW fitting.

Alongside diffusion, studying the *α*‐relaxation process provides us insight into the caged‐to‐diffusive transition in the MSEs, a transition present at all temperatures but more notably at elevated temperatures. Indeed, both FSI‐ and TFSI‐based MSEss show a pronounced temperature dependence in F(Q,t), reflecting changes in ion relaxation dynamics (**Figure** **8**). At 400 K, F(Q,t) decays slowly without reaching zero, indicating strong caging effects that restrict ion motion and prevent complete *α*‐relaxation. This behavior aligns with studies demonstrating that robust Coulombic interactions and long ion‐pair lifetimes dominate at lower temperatures, resulting in constrained ionic dynamics.^[^ ^52^, ^53^, ^54^ ^]^

At 600 K, F(Q,t) decays fully to zero, marking the onset of *α*‐relaxation and diffusive behavior, and for 800 K and 1000 K, this becomes progressively faster. Temperature‐induced reductions in ion‐pair lifetimes and enhanced thermal energy facilitate faster dynamics, as observed for ionic liquid‐based systems.^[^ ^53^, ^55^, ^56^ ^]^

These observations underscore a clear caged‐to‐diffusive transition and to quantify the *α*‐relaxation, we fit the F(Q,t) curves to the Kohlrausch–Williams–Watts (KWW) function, whereby we can extract relaxation times, $\tau_{\alpha }$ (Figure 8c), and stretching exponents, *β* (**Table** **1**).

**Table 1:** Extracted KWW relaxation stretching exponents (*β*) and $R^{2}$ values for both FSI‐ and TFSI‐based MSEs at different temperatures.

Temperature [K]	[Li, Na, K, Ca] FSI		[Li, Na, K, Ca] TFSI	
	*β*	$R^{2}$	*β*	$R^{2}$
400	$0.38\;\pm \;2.17\;\times \;10^{-3}$	0.994	$0.74\;\pm \;1.06\;\times \;10^{-2}$	0.980
600	$0.50\;\pm \;1.05\;\times \;10^{-2}$	0.948	$0.24\;\pm \;5.95\;\times \;10^{-3}$	0.950
800	$0.61\;\pm \;2.62\;\times \;10^{-2}$	0.863	$1.00\;\pm \;9.86\;\times \;10^{-2}$	0.935
1000	$0.93\;\pm \;1.81\;\times \;10^{-1}$	0.897	$0.86\;\pm \;1.59\;\times \;10^{-2}$	0.764

The TFSI‐based MSE consistently exhibits longer relaxation times across all temperatures as compared to the FSI‐based MSE, consistent with findings that steric hindrance and larger molecular structures impose structural constraints that prolong caging effects and extend relaxation times.^[^ ^55^, ^57^ ^]^ These findings highlight the critical role of steric factors and ionic interactions in governing relaxation dynamics.

While correlation coefficients ($R^{2}$) vary across datasets, this variability reflects the inherent dynamic heterogeneity of MSEs, particularly at higher temperatures where increased ion mobility leads to deviations from single‐exponential relaxation.^[^ ^58^ ^]^ Such heterogeneity is a well‐known characteristic of highly concentrated electrolytes, ionic liquids, and glass‐forming materials, where relaxation processes are influenced by a distribution of timescales rather than a single dominant timescale.^[^ ^59^ ^]^ The KWW model remains an appropriate choice for analyzing relaxation dynamics in these systems, as it effectively captures the stretched‐exponential behavior arising from dynamic heterogeneity.^[^ ^60^ ^]^ Additional details, including error handling and statistical validation of the fits, are provided in the SI.

The stretching exponent, *β*, further highlights differences in the relaxation dynamics of the two MSEs. For the FSI‐based MSE, *β* increases with temperature, from 0.50 at 600 K to 0.93 at 1000 K, indicating a shift from more heterogeneous dynamics at lower temperature to more homogeneous, single‐exponential relaxation behavior as the temperature is increased. In contrast, the TFSI‐based MSE shows greater variability in *β*. Notably, at 800 K, *β* = 1.0, indicating nearly single‐exponential (Debye‐like) relaxation—a behavior that deviates from typical KWW dynamics, which generally account for nonuniform relaxation processes. At 1000 K, *β* decreases to 0.86, suggesting a slight shift back toward more heterogeneous dynamics, though it remains close to Debye‐like relaxation. This variability in *β* for TFSI‐based MSE may arise from the bulkier and more flexible TFSI anions, which introduce diverse steric and electrostatic interactions within the local environment. The Supplementary Information (SI) provides comprehensive details on the treatment of errors and uncertainties associated with the reported parameters.

**Figure 9:**
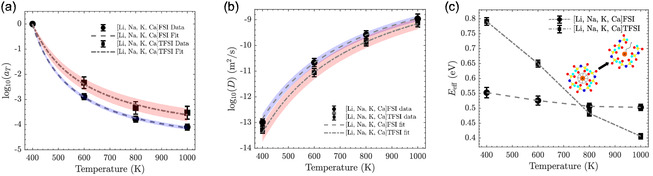
a) WLF fit for [Li, Na, K, Ca] FSI and [Li, Na, K, Ca] TFSI MSEs with 95% confidence intervals (CIs). b) Arrhenius fits for the self‐diffusion coefficients of the MSEs with 95% CIs. c) Effective energy barriers ($E_{\text{eff}}$) for cage escaping as a function of temperature for the MSEs.

The Williams–Landel–Ferry (WLF) fit (**Figure** **9**a) shows the temperature‐dependent shift in relaxation times for both FSI‐ and TFSI‐based MSEs. The shift factor, $a_{T}$, decreases with increasing temperature, consistent with thermally activated relaxation processes. This trend aligns with studies indicating that ionic liquids with TFSI anions exhibit slower relaxation dynamics compared to FSI‐based ones due to steric hindrance and stronger caging effects at lower temperatures.^[^ ^61^, ^62^ ^]^ Here, both MSEs follow similar thermal activation profiles up to 800 K, but from 800 to 1000 K, TFSI shows a somewhat steeper slope, indicating a greater sensitivity to thermal activation at higher temperatures.

The self‐diffusion coefficients were fitted to the Arrhenius equation (Figure 9b), yielding activation energies ($E_{a}$) from the global fit that represent the average energy barriers for ions to escape their cages. These barriers decrease with increasing temperature, reflecting the progressive easing of caging effects and the transition toward diffusive motion. Both electrolytes exhibit this trend of decreasing effective energy barriers ($E_{\text{eff}}=-k_{B}\;d\ln(D)/d(1/T)$) with rising temperature (Figure 9c).

For the FSI‐based MSE, $E_{a}$ is slightly lower than for TFSI, aligning with its faster diffusion rates (**Table** **2**). This suggests that FSI anions create a less restrictive local environment, facilitating ion mobility, as demonstrated in studies comparing FSI and TFSI anions in ionic liquid electrolytes.^[^ ^61^, ^63^ ^]^ The effective energy barrier for FSI remains relatively stable (Figure 9c), decreasing only slightly from ≈0.55 eV at 400 K to ≈0.51 eV at 1000 K, indicating a consistently low‐resistance caging environment.

**Table 2:** Global Arrhenius fit parameters, pre‐exponential factor ($D_{0}$) and activation energy ($E_{a}$), for [Li, Na, K, Ca]FSI and [Li, Na, K, Ca]TFSI.

MSE	$D_{0}$ [m 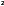 /s]	$E_{a}$ [eV]	$R^{2}$
[Li, Na, K, Ca] FSI	$4.28\times 10^{-7}\pm 2.88\times 10^{-8}$	$5.14\times 10^{-1}\pm 4.76\times 10^{-3}$	0.999
[Li, Na, K, Ca] TFSI	$4.55\times 10^{-7}\pm 6.33\times 10^{-8}$	$5.60\times 10^{-1}\pm 8.49\times 10^{-3}$	0.844

In contrast, the effective energy barrier for TFSI decreases significantly; from ≈0.79 eV at 400 K to ≈0.41 eV at 1000 K, reflecting strong caging effects at lower temperatures that weaken as thermal energy increases.^[^ ^64^ ^]^ Below 800 K, TFSI exhibits higher energy barriers compared to FSI, likely due to its bulkier, sterically hindered structure.^[^ ^62^ ^]^ However, beyond 800 K, TFSI barriers drop below those of FSI, suggesting reduced resistance to ion mobility at higher temperatures. The SI provides comprehensive details on the treatment of errors and uncertainties associated with the reported parameters.

This crossover at 800 K may be attributed to conformational or dynamic changes in TFSI at elevated temperatures, which reduce steric hindrance and lower the energy barriers. In contrast, FSI, with its less sterically hindered structure, does not undergo similar relaxation, leading to relatively stable $E_{\text{eff}}$ values.^[^ ^61^, ^64^ ^]^

The observed temperature dependence highlights a clear caged‐to‐diffusive transition across the studied temperature range (400–1000 K). At lower temperatures, strong Coulombic interactions result in pronounced caging effects, limiting the mobility of ${\text{Ca}}^{2+}$. As the temperature increases, thermal energy allows ions to escape their cages, enabling them to overcome energy barriers and achieve higher diffusion rates. Notably, the FSI‐based MSEs exhibit higher mobility, attributed to less steric hindrance. Interestingly, a significant reduction in energy barriers is observed for TFSI‐based MSEs at temperatures exceeding 800 K, suggesting a dynamic restructuring of the local ionic environment. These findings underscore the critical role of thermal effects in tuning the macroscopic properties of MSEs and highlight potential pathways to optimize ${\text{Ca}}^{2+}$ transport through temperature modulation.

## 3 Conclusion

The ionic mobility is significantly influenced by complex ion interactions, packing structures, and caging effects, with notable dynamic heterogeneity observed across different ions and temperatures. Strong cation–anion interactions, particularly for ${\text{Ca}}^{2+}$, lead to restricted ion mobility due to the formation of tight coordination “shells” that limit movement. This effect is more pronounced in the TFSI‐based MSE, where lower cation CNs lead to stronger cages.

The temperature‐dependent trends suggest a viable pathway to enhance the ion mobility: raising the operating temperature to diminish Coulombic interactions would facilitate faster diffusion, a strategy which indeed is possible in practice by virtue of the MSEs being solvent‐free and thermally stable.^[^ ^24^ ^]^

To further enhance the ion mobility in these electrolytes, minute amounts of solvents could be added to weaken the Coulombic interactions, admittedly at the expense of some safety aspects. Diluents, i.e., nonsolvents, could alternatively be added to reduce the overall viscosity while retaining the local structure with strong cation‐anion coordination,^[^ ^65^ ^]^ with the same caveat. Alternative anions could, of course, be explored, especially those with flexible modes of coordination to adjust the local environment around the ${\text{Ca}}^{2+}$ ions, a strategy harder to visualize but with fundamentally larger promises of retained safety.

The insights gained highlight both the challenges and opportunities in designing MSEs for practical applications and show how molecular‐level modifications can yield improved electrolyte performance, particularly for CaBs.

## 4 Experimental Section

4.1

4.1.1

MD simulations were performed using the LAMMPS software package,^[^ ^66^ ^]^ employing the CL&Pol polarizable force field.^[^ ^67^, ^68^, ^69^, ^70^ ^]^ The validation of the force field, including comparisons of CNs and self‐diffusion coefficients with literature, was detailed in the SI. Cubic simulation boxes containing 1575 ions (175 of each cation (${\text{Li}}^{+}$, ${\text{Na}}^{+}$, $K^{+}$, and ${\text{Ca}}^{2+}$) and 875 anions (FSI or TFSI)) were created using the *Packmol* software.^[^ ^71^ ^]^ The molecular topology files, Lennard–Jones parameters, and bonded parameters were generated using the *fftool* package.^[^ ^72^ ^]^

First, an energy minimization using the conjugate gradients method was made, followed by equilibration runs at four different temperatures: 400, 600, 800, and 1000 K. For each temperature, an equilibration run was performed in the isothermal‐isobaric ensemble (NPT) at 1 atm for 3 ns, followed by equilibration in the canonical ensemble (NVT) for 3 ns, and a further 5 ns equilibration in the NVT ensemble.

The temperature range was selected to explore the temperature‐dependent structural and dynamic behaviors of MSEs. The thermal stability of FSI‐ and TFSI‐based salts was well‐documented in the literature, with TFSI systems exhibiting higher stability. For instance, ionic liquids containing LiTFSI were reported to be stable up to 600–650 K,^[^ ^73^ ^]^ while those with LiFSI decompose at slightly lower temperatures (500–550 K).^[^ ^74^ ^]^ Regarding potassium salts, studies indicate that KFSI decomposes at temperatures above 473 K, whereas KTFSI exhibits higher thermal stability, with decomposition temperatures exceeding 673 K.^[^ ^75^ ^]^ Recent studies on calcium salts confirm similar trends, with ${\text{Ca(TFSI)}}_{2}$ and ${\text{Ca(FSI)}}_{2}$ exhibiting thermal decomposition temperatures of ≈600–650 K and 500–550 K, respectively.^[^ ^24^ ^]^ Specific thermal stability data for sodium salts (NaFSI and NaTFSI) are less readily available. However, given the trends observed for lithium and potassium salts, it is reasonable to hypothesize that sodium salts may exhibit similar thermal behaviors, with TFSI‐based systems demonstrating higher stability. In general, TFSI‐based salts tend to have higher thermal stability compared to FSI‐based salts across different cations.

The simulations conducted at 800 K and 1000 K exceed the exact thermal stability of the individual salts but were intentionally chosen to accelerate ion dynamics and gain insights into fundamental transport mechanisms, such as caged‐to‐diffusive transitions and structural rearrangements. High‐temperature simulations are commonly employed in MD studies of ionic liquids and molten salts to investigate long‐term dynamical behavior and ion mobility within practical computational timescales. Such simulations allow us to capture phenomena that would require significantly longer simulation times at lower temperatures, thereby providing a comprehensive understanding of the transport properties of these electrolytes.

The production runs, using the average simulation box size obtained from the NPT equilibrium runs, were conducted in the NVT ensemble for different durations depending on the temperature: 400 K = 70 ns, and 600, 800, and 1000 K = 40 ns each. A Nosé–Hoover thermostat with a temperature damping constant of 100 fs and a pressure damping constant of 1000 fs was applied. Electrostatic interactions were computed using the PPPM scheme, and periodic boundary conditions were implemented in all directions.

Structural analysis, by RDFs and CNs (CNs), was performed using LAMMPS subroutines. The static structure factor (S(Q))^[^ ^76^, ^77^ ^]^ was computed using a custom MATLAB script. Dynamics analysis involved computing self‐diffusion coefficients,^[^ ^78^ ^]^ the intermediate scattering function (F(Q,t)),^[^ ^79^ ^]^ and relaxation times. The F(Q,t) curves were fit to the Kohlrausch–Williams–Watts (KWW) function,^[^ ^80^, ^81^ ^]^ and the resulting relaxation times were analyzed with the WLF equation^[^ ^82^ ^]^ to capture temperature dependence. Additionally, the self‐diffusion coefficients were fitted to the Arrhenius equation to estimate energy barriers associated with ion cage escape.^[^ ^83^ ^]^ The Arrhenius model was selected due to its suitability for describing thermally activated processes over the studied temperature range.

## Conflict of Interest

The authors declare no conflict of interest.

## Supporting information

Supplementary Material

## Data Availability

The data that support the findings of this study are available from the corresponding author upon reasonable request.
