# Supplementary material for: Local Structure and Dynamics in Solvent‐Free Molten Salt Ca2+‐Electrolytes
Source: Chemphyschem. 2025 Jun 24;26(15):e202500090. doi: 10.1002/cphc.202500090 (PMC12321286; doi:10.1002/cphc.202500090)
Supplement: Supplementary file 1 — Supplementary Material [file CPHC-26-e202500090-s001.pdf]

Supplementary Information to: " Local Structure and Dynamics in Solvent-Free Molten Salt Ca^{2+}-Electrolytes"

Carolina Cruz[1] and Patrik Johansson[1,2,3]

[1]*Department of Physics, Chalmers University of Technology, 412 96 Göteborg, Sweden*

[2]*Alistore-ERI, CNRS FR 3104, 15 Rue Baudelocque, 80039 Amiens, France*

[3]*Department of Chemistry–Ångström, Uppsala University, SE-751 21 Uppsala, Sweden*

S1. REPRESENTATIVE LAMMPS INPUT FILE

```
# LAMMPS Script for Production - RDF Calculation
# ----------------------------------------------------------
# Stage 1: Initialize LAMMPS run for 3-d periodic
# ----------------------------------------------------------
echo            both
units           real
boundary        p p p
atom_style      full
bond_style      harmonic
angle_style     harmonic
dihedral_style  opls
special_bonds   lj/coul 0.0 0.0 0.5
pair_style      hybrid/overlay lj/cut/coul/long 10.0 12.0 coul/long
   /cs 12.0 thole 2.600 12.0 coul/tt 4 12.0
pair_modify     mix geometric tail yes
kspace_style    pppm 1.0e-4
# ------ Read initial configuration - file with Drude oscillators
   added
    read_restart data-peq.mix
# ------ Read pair interactions involving Drude particles
    include pair-sc.lmp
    include pair-drude.lmp
    include pair-tt.lmp
# --- Groups
group      ATOMS         type 1 2 3 4 5 6 7 8
group      CORES         type 2 3 4 5
group      DRUDES        type 9 10 11 12
group      cation_Li     type  1
group      cation_Na     type  6
group      cation_K      type  7
group      cation_Ca     type  8
group      anion         type  2 3 4 5
group      CaFSI         union   cation_Ca anion
group      LiFSI         union   cation_Li anion
```

```
group       NaFSI         union    cation_Na anion
group       KFSI          union    cation_K anion
# -----------------------------------------------------------
fix DRUDE all drude N C C C C N N N D D D D
# identify each atom type: [C]ore, [D]rude, [N]on-polarizable
# -----------------------------------------------------------
#   Variables
# -----------------------------------------------------------
variable     tstep     equal   1.0
variable     TK        equal   1000.0
variable     TDRUDE    equal   1.0
variable     PBAR      equal   1.0
variable     Nf        equal   10
variable     Ndump     equal   1000
variable     Tdamp     equal   100*${tstep}
variable     Pdamp     equal   1000*${tstep}
variable     Nf        equal   10
variable     Ne        equal   1
variable     Nr        equal   ${Nf}/${Ne}
variable     Nrunp   equal   5E6
variable     Nrund   equal   10E6
variable     Nr_rdf  equal   ${Nrund}/${Nf}
variable     N_run   equal   1
#-----------------------------------------------------------------------------------------
# Stage 2: Minimization
#-----------------------------------------------------------------------------------------
log               Minimization.dat
min_style         cg
minimize          1.0e-4 1.0e-6 100 5000
reset_timestep    0
#-----------------------------------------------------------------------------------------
# Stage 3: Set the initial velocities for $T
#-----------------------------------------------------------------------------------------
neighbor          2.0 bin
neigh_modify      delay 0 every 1 check yes
timestep          ${tstep}
#-----------------------------------------------------------------------------------------
# Stage 4: NVT Production
#-----------------------------------------------------------------------------------------
compute TDRUDE all temp/drude
log             Production_NVT_${N_run}.dat
```

```
fix             TSTAT all tgnvt/drude temp ${TK} ${TK} ${Tdamp} ${TDRUDE} 20
thermo_style    custom step time cpu etotal ke pe ebond eangle evdwl ecoul elong press vol density f_TSTAT[1] f_TSTAT[2] f_TSTAT[3]
thermo_modify   flush yes
thermo          ${Nf}
run             ${Nrunp}
# ---- Write restart file
write_restart   restart-prod.mix
#-------------------------------------------------------------------------------------------------
# Stage 5: RDF
#-------------------------------------------------------------------------------------------------
log                 Production_RDF.dat
thermo_style        custom time temp etotal density vol press
thermo_modify       flush yes
thermo              ${Nf}
# --- Radial distribution function per interaction pairs
compute         myRDF all rdf 500  1 1   1 2  1 3  1 4  1 5   6 6   6 2  6 3  6 4  6 5   7 7  7 2  7 3  7 4  7 5   8 8  8 2  8 3  8 4  8 5
fix             RDF all ave/time ${Ne} ${Nr_rdf} ${Nrund} c_myRDF[*] file RDF.dat mode vector
# --- Total Radial distribution function
compute         myRDF_tot all rdf 500
fix             RDF_tot all ave/time ${Ne} ${Nr_rdf} ${Nrund} c_myRDF_tot[*] file RDF_tot.dat mode vector
# --- Radial distribution function salts
compute         myRDF1 CaFSI rdf 500
fix             RDF1 CaFSI ave/time ${Ne} ${Nr_rdf} ${Nrund} c_myRDF1[*] file RDF_CaFSI.dat mode vector

compute         myRDF2 LiFSI rdf 500
fix             RDF2 LiFSI ave/time ${Ne} ${Nr_rdf} ${Nrund} c_myRDF2[*] file RDF_LiFSI.dat mode vector

compute         myRDF3 NaFSI rdf 500
fix             RDF3 NaFSI ave/time ${Ne} ${Nr_rdf} ${Nrund} c_myRDF3[*] file RDF_NaFSI.dat mode vector

compute         myRDF4 KFSI rdf 500
fix             RDF4 KFSI ave/time ${Ne} ${Nr_rdf} ${Nrund} c_myRDF4[*] file RDF_KFSI.dat mode vector

run             ${Nrund}
# ---- Write restart file
write_restart   restart-rdf.mix
```

S2. FORCE FIELD VALIDATION

To ensure the reliability of the CL&Pol polarizable force field in simulating MSEs, we validated our simulations by comparing calculated properties against reported literature values for similar salts. Specifically, coordination numbers from our simulations were benchmarked against studies on ionic liquids and highly concentrated electrolytes (Table S1).

TABLE S1: Comparison of reported and simulated coordination numbers (CN) using various force fields for ionic liquid and electrolyte systems.

Electrolyte	Reported CN	Temperature (K)	Force Field	Simulated CN	Source
LiTFSI in [emim][TFSI]	4.2	423	APPLE&P	4.3	[1]
LiTFSI in [emim][TFSI]	4.63		Modified APPLE&P		
NaTFSI in [emim][TFSI]	5.5	333	DP-FF (Polarizable)	5.4	[2]
NaTFSI in [emim][TFSI]	6.0		NP-FF (Non-polarizable)		
KTFSI in PC	6.0	298	OPLS-AA	7.2	[3]
KFSI in DME	7.5	298	OPLS-AA		[4]

Additionally, we replicated previously reported simulations using the respective computational approaches but implementing the CL&Pol force field. The validation focused on comparing coordination numbers and self-diffusion coefficients, which are critical metrics for assessing the accuracy of the force field.

For $Ca(TFSI)_2$ in DMF, [5] used the PCFF+ force field and reported partial coordination numbers of Ca-O(TFSI) as 1.5 and Ca-N(TFSI) as 1.8, with a self-diffusion coefficient of approximately 5×10^{-10} m^2/s at 298 K. Using the CL&Pol force field for this electrolyte, we obtained partial coordination numbers of Ca-O(TFSI) as 2.1 and Ca-N(TFSI) as 1.7, with a self-diffusion coefficient of 1.1×10^{-10} m^2/s (Figure S1).

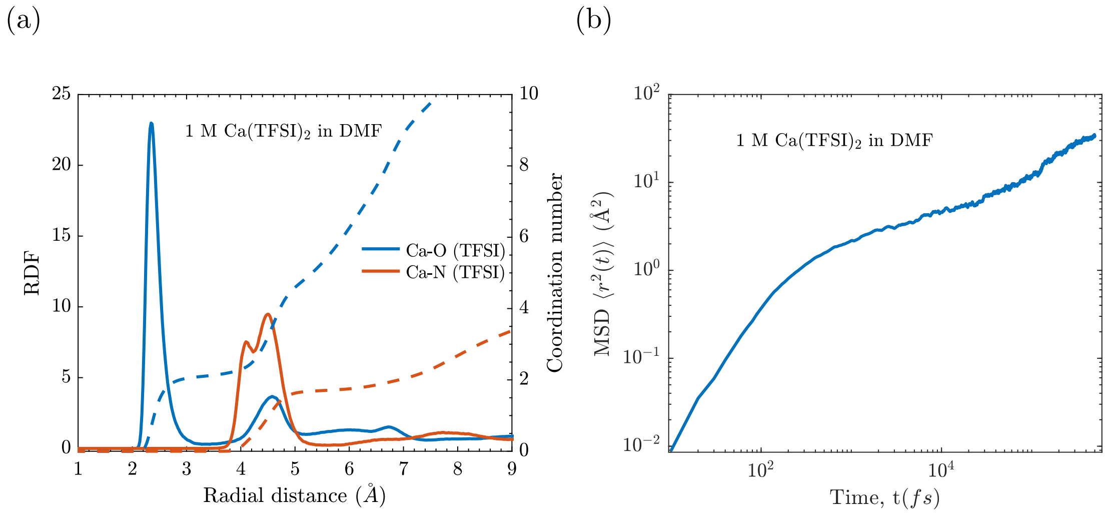

FIG. S1: (a) Radial distribution function (RDF) and coordination number, (b) mean squared displacement (MSD) for a 1 M solution of $Ca(TFSI)_2$ in DMF at 298 K.

Bouazizi *et al.* [6] used the OPLS-AA force field to study a 1 mol/kg solution of $LiPF_6$ in DMSO. The authors reported a coordination environment dominated by approximately 4 DMSO molecules around each Li^+ and a self-diffusion coefficient of 1.6×10^{-10} m^2/s. Using the CL&Pol force field, we obtained a coordination number of Li-O(DMSO) as 5 and a self-diffusion coefficient of 1.63×10^{-10} m^2/s (Figure S2).

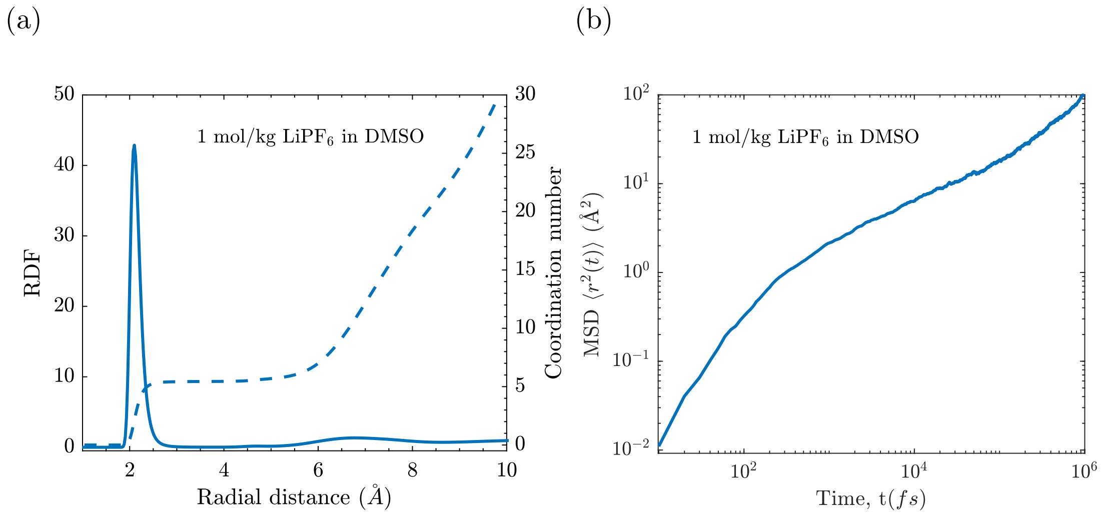

FIG. S2: (a) Radial distribution function (RDF) and coordination number, (b) mean squared displacement (MSD) for a 1 M solution of $LiPF_6$ in DMSO.

S3. RADIAL DISTRIBUTION FUNCTIONS

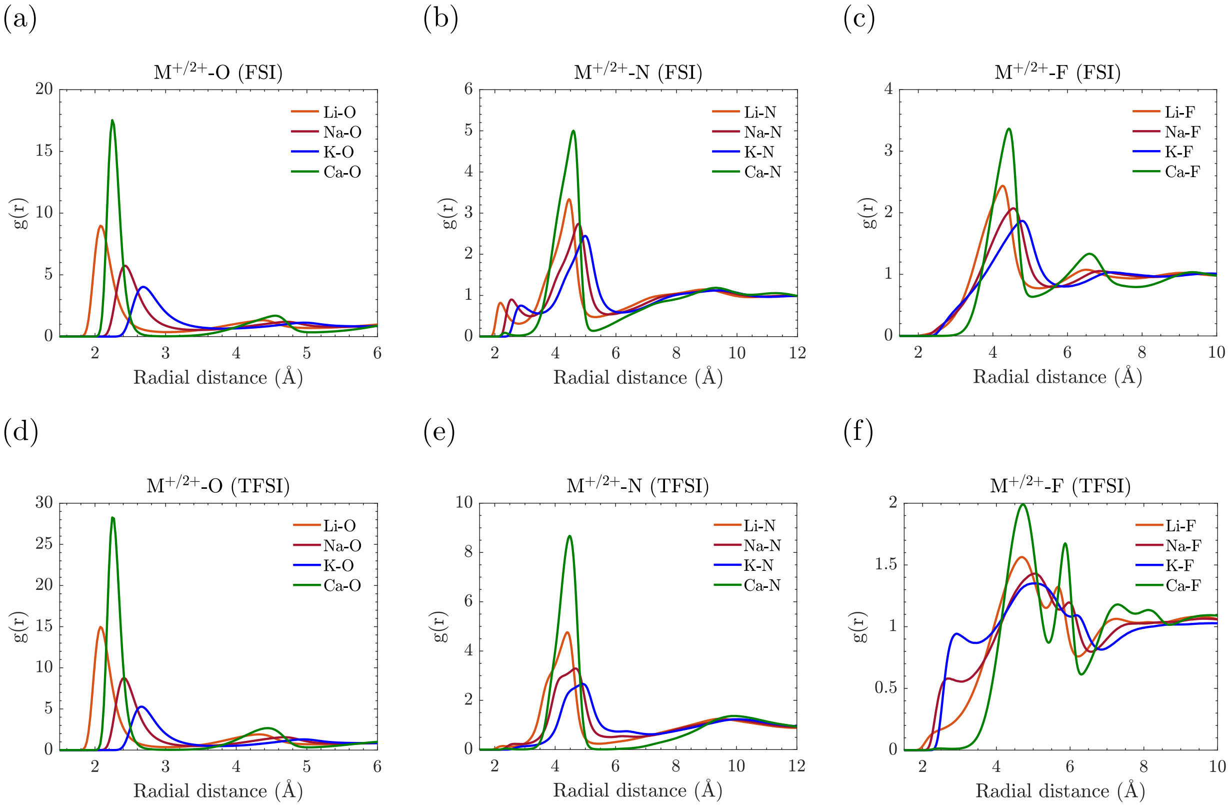

FIG. S3: Radial distribution functions, $g(r)$, at T = 600 K for $M^{+/2+}$-O, $M^{+/2+}$-N, and $M^{+/2+}$-F interactions in FSI-based (a–c) and TFSI-based (d–f) MSs, where $M^{+/2+}$ represents Li^{+}, Na^{+}, K^{+}, and Ca^{2+} cations.

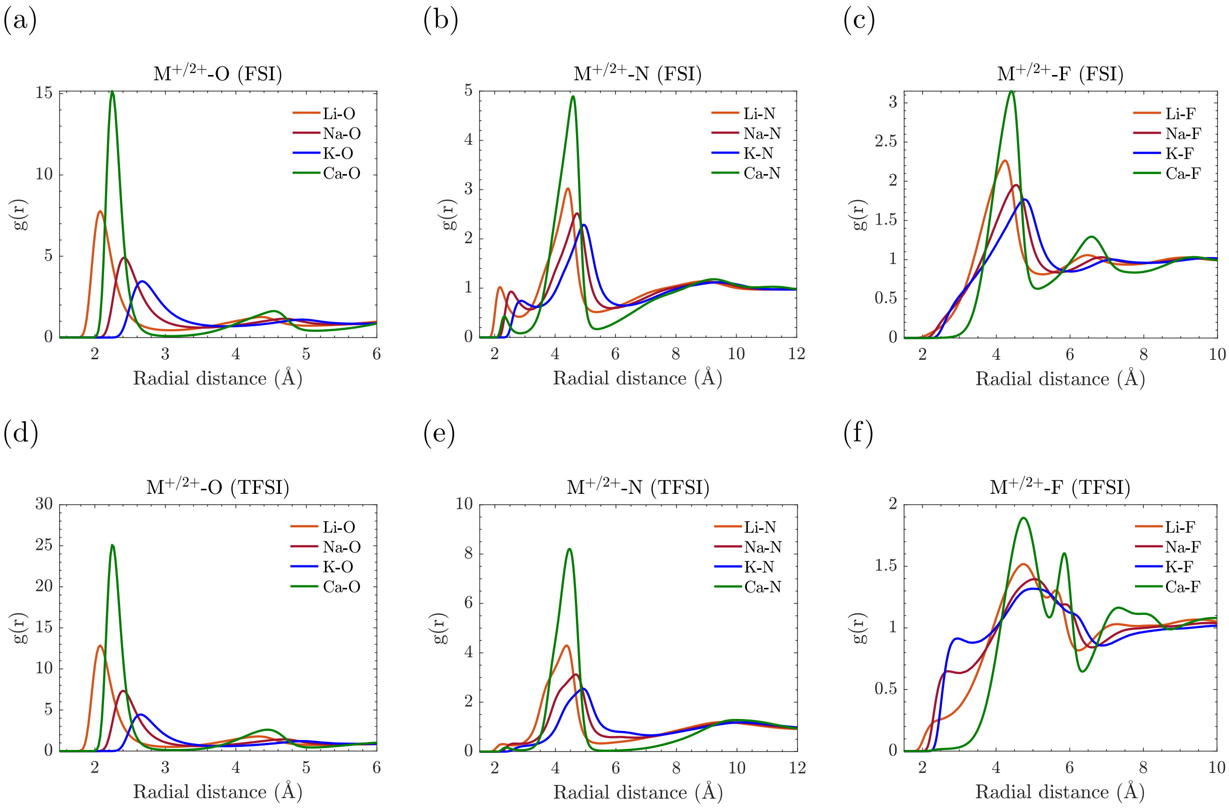

FIG. S4: Radial distribution functions, $g(r)$, at T = 800 K for $M^{+/2+}$-O, $M^{+/2+}$-N, and $M^{+/2+}$-F interactions in FSI-based (a–c) and TFSI-based (d–f) MSs, where $M^{+/2+}$ represents Li^{+}, Na^{+}, K^{+}, and Ca^{2+} cations.

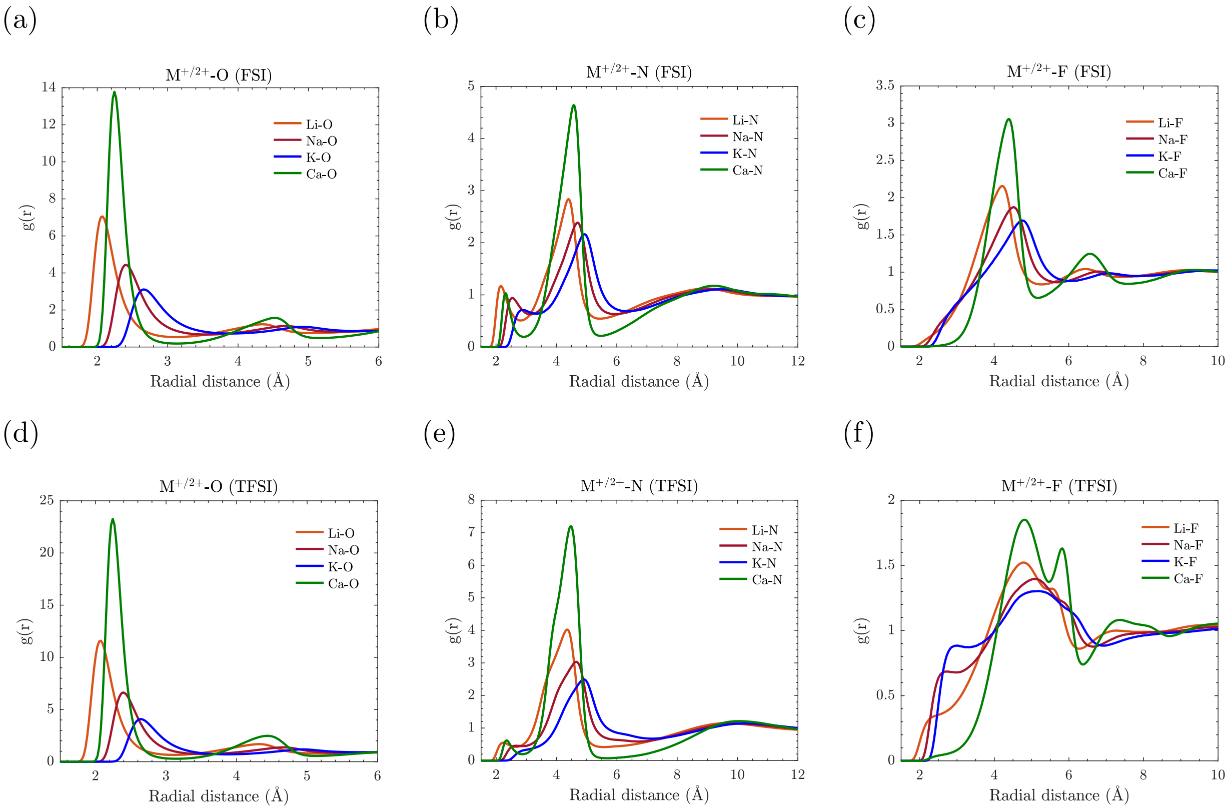

FIG. S5: Radial distribution functions, $g(r)$, at T = 1000 K for $M^{+/2+}$-O, $M^{+/2+}$-N, and $M^{+/2+}$-F interactions in FSI-based (a–c) and TFSI-based (d–f) MSs, where $M^{+/2+}$ represents Li^{+}, Na^{+}, K^{+}, and Ca^{2+} cations.

S4. TOTAL COORDINATION NUMBERS

TABLE S2: CNs of the FSI- and TFSI-based MSEs at 400, 600, 800, and 1000 K.

	400 K		600 K		800 K		1000 K	
	FSI	TFSI	FSI	TFSI	FSI	TFSI	FSI	TFSI
Li	5.0	4.3	4.8	4.3	4.6	4.0	4.4	3.7
Na	6.0	5.4	5.9	5.3	5.6	4.8	5.2	4.6
K	7.2	6.6	6.7	5.8	6.2	5.4	5.7	4.9
Ca	6.0	6.0	6.0	6.0	5.9	5.9	5.9	5.6

S5. PARTIAL COORDINATION NUMBERS

TABLE S3: Partial coordination numbers of the FSI-based MSE at 400 K

Pairwise	Li	Na	K	Ca
O	4.79	5.71	6.73	5.98
N	0.20	0.34	0.48	0.05
F	0.00	0.00	0.00	0.00

TABLE S4: Partial coordination numbers of the TFSI-based MSE at 400 K

Pairwise	Li	Na	K	Ca
O	4.26	4.49	4.22	5.88
N	0.20	0.05	0.00	0.03
F	0.00	0.90	2.39	0.05

TABLE S5: Partial coordination numbers of the FSI-based MSE at 600 K

Pairwise	Li	Na	K	Ca
O	4.60	5.53	6.29	6.00
N	0.18	0.33	0.38	0.01
F	0.00	0.00	0.00	0.00

TABLE S6: Partial coordination numbers of the TFSI-based MSE at 600 K

Pairwise	Li	Na	K	Ca
O	4.24	4.38	4.01	5.97
N	0.02	0.03	0.00	0.00
F	0.00	0.77	1.84	0.01

TABLE S7: Partial coordination numbers of the FSI-based MSE at 800 K

Pairwise	Li	Na	K	Ca
O	4.34	5.22	5.86	5.93
N	0.23	0.34	0.35	0.07
F	0.00	0.00	0.00	0.00

TABLE S8: Partial coordination numbers of the TFSI-based MSE at 800 K

Pairwise	Li	Na	K	Ca
O	3.98	3.99	3.79	5.85
N	0.04	0.05	0.00	0.02
F	0.00	0.80	1.66	0.00

TABLE S9: Partial coordination numbers of the FSI-based MSE at 1000 K

Pairwise	Li	Na	K	Ca
O	4.13	4.91	5.38	5.71
N	0.25	0.32	0.29	0.16
F	0.00	0.00	0.00	0.00

TABLE S10: Partial coordination numbers of the TFSI-based MSE at 1000 K

Pairwise	Li	Na	K	Ca
O	3.67	3.76	3.55	5.50
N	0.06	0.05	0.00	0.06
F	0.00	0.92	1.65	0.00

S6. KWW FITTING

The fitting of the intermediate scattering function (ISF) data was performed using the Kohlrausch-Williams-Watts (KWW) stretched exponential function:

$$F(Q,t) = A\exp\left[-\left(\frac{t}{\tau_\alpha}\right)^\beta\right] \tag{S1}$$

where A is the amplitude, τ_α is the alpha relaxation time, and β is the stretching exponent. This functional form was fitted to the ISF data at each temperature using a nonlinear least squares fitting, which was implemented via the `lsqcurvefit` function in MATLAB.

The fit quality was assessed by minimizing the residual sum of squares between the fitted model and the data. Uncertainties in the fitted parameters were quantified using the Jacobian matrix (J) generated during the fitting process. The parameter covariance matrix (Σ) was calculated as:

$$\Sigma = (J^\top J)^{-1} \cdot \frac{RSS}{\nu} \tag{S2}$$

where RSS is the residual sum of squares, and ν is the degrees of freedom in the fit. The diagonal elements of Σ correspond to the variances of the fitted parameters.

To estimate the 95% confidence intervals for the parameters, the following equation was used:

$$CI = t_{\nu,1-\alpha/2} \cdot \sqrt{diag(\Sigma)} \tag{S3}$$

where $t_{\nu,1-\alpha/2}$ is the critical value from the Student's t-distribution for the specified confidence level and ν degrees of freedom.

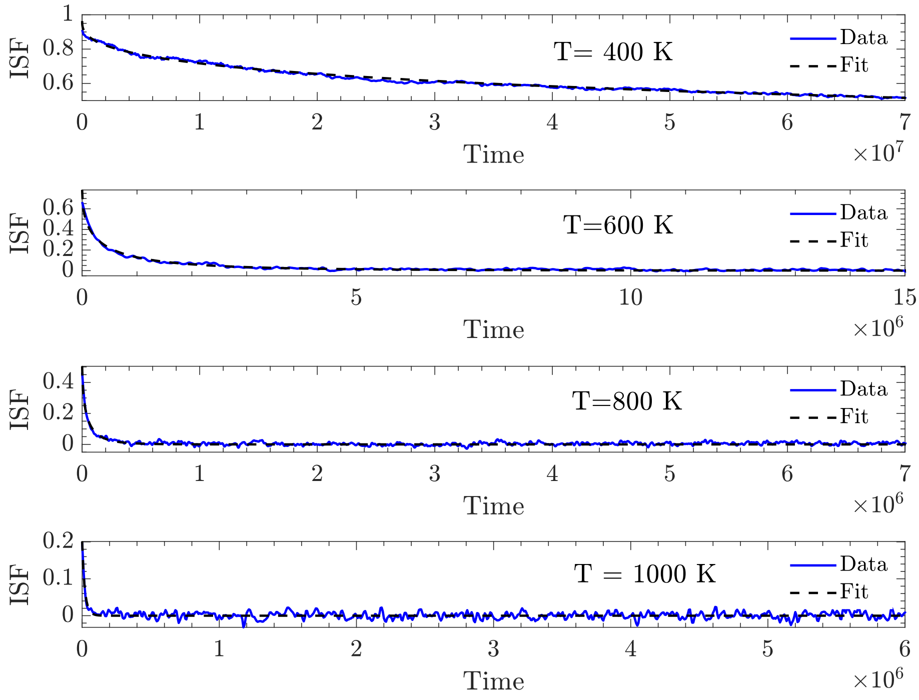

FIG. S6: KWW fitting of the ISF for the FSI-based MSE at 400, 600, 800, and 1000 K.

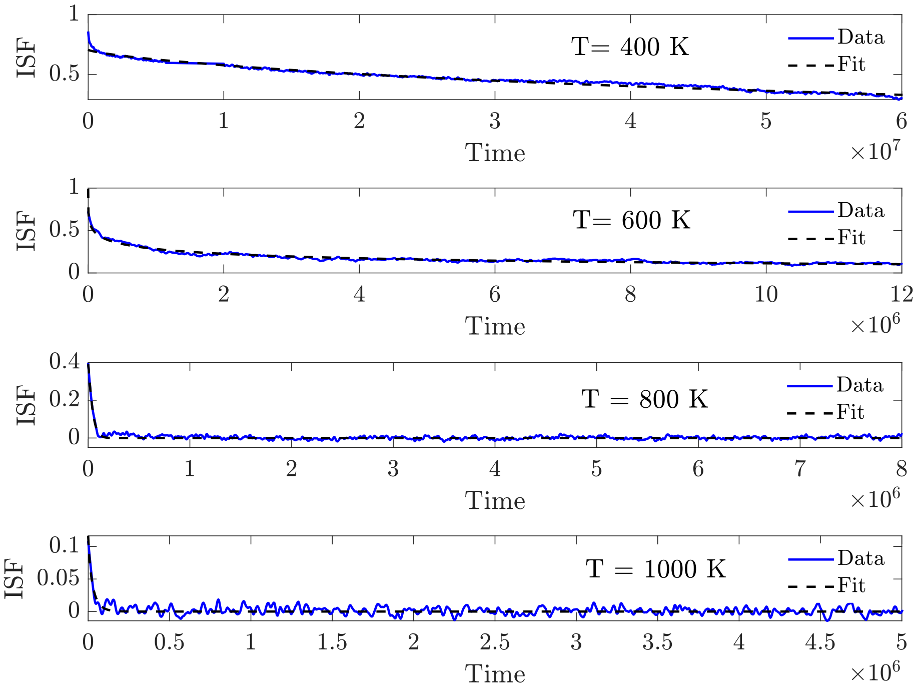

FIG. S7: KWW fitting of the ISF for the TFSI-based MSE at 400, 600, 800, and 1000 K.

S7. WLF FITTING

The Williams-Landel-Ferry (WLF) equation was employed to model the shift factors:

$$\log_{10} a_T = -C_1 \frac{T - T_{ref}}{C_2 + (T - T_{ref})} \tag{S4}$$

where C_1 and C_2 are the fit parameters representing the sensitivity of the relaxation behavior to temperature deviations from $T_{ref} = 400K$.

The MATLAB `lsqcurvefit` function was used to fit the WLF model to the logarithmic shift factors. The fit quality was assessed by analyzing the residuals and R^2 values. Bootstrap resampling was performed to quantify the uncertainties in the fit parameters C_1 and C_2. Residuals were resampled with replacement, and the WLF model was refitted 1000 times to generate distributions for C_1 and C_2. The standard deviations of these distributions were used to estimate the uncertainties. Vertical error bars for the shift factor data points were derived from the sensitivity of the fit parameters. Additionally, 95% confidence intervals for the WLF fit curve were calculated using the bootstrap results. The confidence intervals were visualized as shaded regions around the fit curve.

TABLE S11: WLF fitting parameters, C_1 and C_2, and coefficients of determination (R^2) for the FSI- and TFSI-based MSEs.

MSE	C_1	C_2	R^2
[Li, Na, K, Ca]FSI	5.220 ±0.073	159.365 ±7.532	0.999
[Li, Na, K, Ca]TFSI	4.836 ±60.268	204.998 ±33.454	0.997

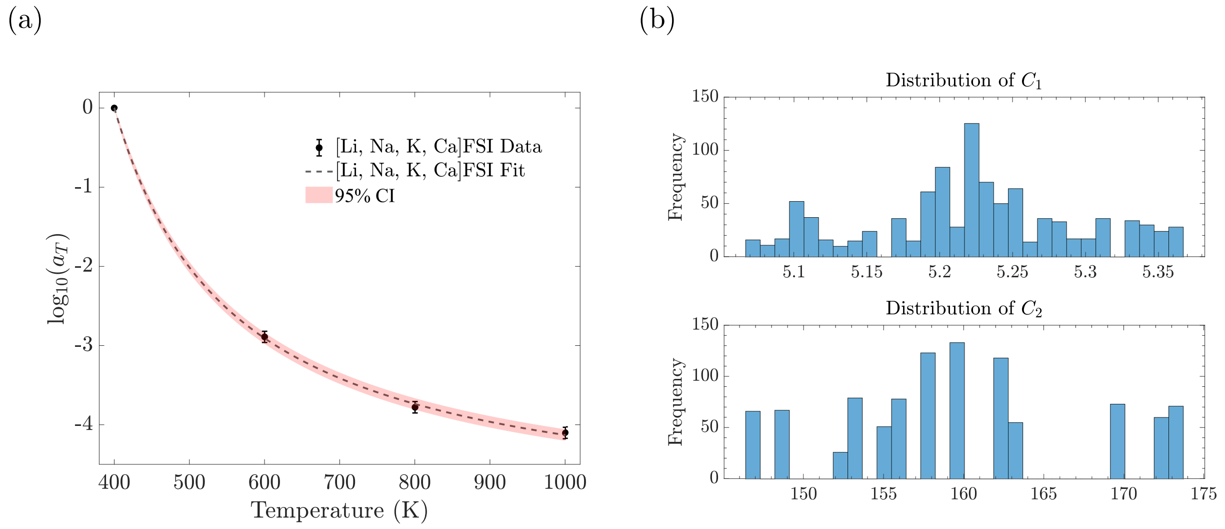

FIG. S8: (a) WLF fitting of the shift factor (a_T) with a 95% confidence interval (shaded region) for the FSI-based MSE. (b) Distributions of the WLF fitting parameters C_1 (top) and C_2 (bottom), obtained through bootstrap resampling of residuals.

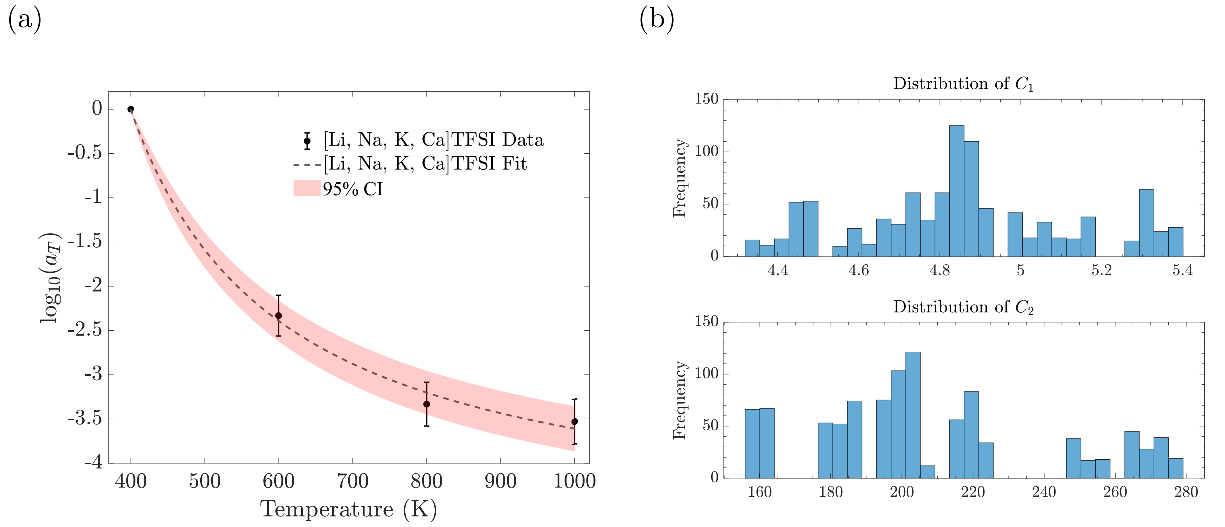

FIG. S9: (a) WLF fitting of the shift factor (a_T) with a 95% confidence interval (shaded region) for the TFSI-based MSE. (b) Distributions of the WLF fitting parameters C_1 (top) and C_2 (bottom), obtained through bootstrap resampling of residuals.

S6. COORDINATION MODES

	400 K				600 K				800 K			
	FSI		TFSI		FSI		TFSI		FSI		TFSI	
	Possible SN	Possible coordination modes	Possibe SN	Possible coordination modes	Possible SN	Possible coordination modes	Possible SN	Possible coordination modes	Possible SN	Possible coordination modes	Possible SN	Possible coordination modes
Li	5	5 monodentate	4	4 monodentate	5	5 monodentate	4	4 monodentate	5	5 monodentate	4	4 monodentate
	3	2 bidentate + 1 monodentate	3	1 bidentate + 2 monodentate	3	2 bidentate + 1 monodentate	3	1 bidentate + 2 monodentate	3	2 bidentate + 1 monodentate	3	1 bidentate + 2 monodentate
	4	1 bidentate + 3 monodentate	2	2 bidentate	4	1 bidentate + 3 monodentate	2	2 bidentate	4	1 bidentate + 3 monodentate	2	2 bidentate
Na	6	6 monodentate	5	5 monodentate	6	6 monodentate	5	5 monodentate	6	6 monodentate	5	5 monodentate
	4	2 bidentate + 2 monodentate	4	1 bidentate + 3 monodentate	4	2 bidentate + 2 monodentate	4	1 bidentate + 3 monodentate	5	1 bidentate + 4 monodentate	3	2 bidentate + 1 monodentate
	3	3 bidentate	3	2 bidentate + 1 monodentate	3	3 bidentate	3	2 bidentate + 1 monodentate	4	2 bidentate + 2 monodentate	4	1 bidentate + 3 monodentate
	5	1 bidentate + 4 monodentate	3	3 bidentate	5	1 bidentate + 4 monodentate			3	3 bidentate		
K	7	7 monodentate	7	7 monodentate	7	7 monodentate	6	6 monodentate	6	6 monodentate	5	5 monodentate
	4	3 bidentate + 1 monodentate	4	3 bidentate + 1 monodentate	4	3 bidentate + 1 monodentate	5	1 bidentate + 4 monodentate	5	1 bidentate + 4 monodentate	3	2 bidentate + 1 monodentate
	5	2 bidentate + 3 monodentate	4	2 bidentate + 2 monodentate	5	2 bidentate + 3 monodentate	4	2 bidentate + 2 monodentate	4	2 bidentate + 2 monodentate	4	1 bidentate + 3 monodentate
	6	1 bidentate + 5 monodentate	5	1 bidentate + 4 monodentate	6	1 bidentate + 5 monodentate	3	3 bidentate	3	3 bidentate		
Ca	6	6 monodentate	6	6 monodentate	6	6 monodentate	6	6 monodentate	6	6 monodentate	6	6 monodentate
	3	3 bidentate	5	1 bidentate + 4 monodentate	5	1 bidentate + 4 monodentate	5	1 bidentate + 4 monodentate	5	1 bidentate + 4 monodentate	5	1 bidentate + 4 monodentate
	4	2 bidentate + 2 monodentate	4	2 bidentate + 2 monodentate	4	2 bidentate + 2 monodentate	4	2 bidentate + 2 monodentate	4	2 bidentate + 2 monodentate	4	2 bidentate + 2 monodentate
	5	1 bidentate + 4 monodentate	3	3 bidentate	3	3 bidentate	3	3 bidentate	3	3 bidentate	3	3 bidentate

FIG. S10: Possible solvation numbers (SNs) based on various combinations of coordination modes for a given coordination number (CN).

REFERENCES

[1] V. Lesch, Z. Li, D. Bedrov, O. Borodin, and A. Heuer, The influence of cations on lithium ion coordination and transport in ionic liquid electrolytes: a MD simulation study, Physical Chemistry Chemical Physics **18**, 382 (2016).

[2] P. Kubisiak, P. Wróbel, and A. Eilmes, Molecular Dynamics Investigation of Correlations in Ion Transport in MeTFSI/EMIM–TFSI (Me = Li, Na) Electrolytes, The Journal of Physical Chemistry B **124**, 413 (2020).

[3] S. Faraezi, F. Sharmin, A. Barua, A. M. M. Hasan, T. Ferdaus, and M. S. Khan, Cation-Specific interfacial behavior in organic electrolytes for enhanced energy storage, Electrochimica Acta **508**, 145229 (2024).

[4] J. Li, Y. Hu, H. Xie, J. Peng, L. Fan, J. Zhou, and B. Lu, Weak Cation–Solvent Interactions in Ether-Based Electrolytes Stabilizing Potassium-ion Batteries, Angewandte Chemie International Edition **61**, 10.1002/anie.202208291 (2022).

[5] S. Biria, S. Pathreeker, and I. D. Hosein, A computational study on the ca^{2+} solvation, coordination environment, and mobility in electrolytes for calcium ion batteries, ChemRxiv 10.26434/chemrxiv-2021-614996f (2021), preprint: 10.26434/chemrxiv-2021-614996f.

[6] S. Bouazizi, S. Nasr, and M. C. Bellissent-Funel, MD Simulation and Analysis of the Pair Correlation Functions, Self-Diffusion Coefficients and Orientational Correlation Times in Aqueous KCl Solutions at Different Temperatures and Concentrations, Journal of Solution Chemistry **53**, 1107 (2024).
